# Orthodontic Implants: Novelty and Evolution in Veterinary Orthodontics—Retrospective Case Series Report

**DOI:** 10.3390/vetsci12121169

**Published:** 2025-12-09

**Authors:** Raluca-Ioana Nedelea, Mihai Marian Borzan, Cristinel Cezar Mătură, Ioan Marcus

**Affiliations:** 1 Pathophysiology Department, University of Agricultural Studies and Veterinary Medicine, 400372 Cluj-Napoca, Romania; 2 Animal Breeding and Animal Productions Department, University of Agricultural Studies and Veterinary Medicine, 400372 Cluj-Napoca, Romania; 3 DI-VET Medical, 011635 Bucharest, Romania

**Keywords:** veterinary orthodontics, orthodontic implants, dogs, cats, veterinary dentistry

## Abstract

Orthodontic implants (OIs) are endosseous devices designed to provide stable skeletal anchorage in orthodontic treatment. A variety of terms have been proposed for these devices; however, such terminology may lead to OIs being confused with conventional dental implants. Therefore, the similarities and distinctions between OIs and dental implants will be examined in this research. Three representative clinical cases will be presented, illustrating the application of OIs for mandibular incisor teeth lingualizations, distalization of a maxillary canine tooth, and palatal flap protection for palatoschizis closure in a cat, describing a custom-made protection shield held in place with four OIs. This article will cover the criteria for OI selection, the determination of optimal insertion sites, and the placement technique. Given that a key prerequisite for a successful outcome is primary stability, several complications may occur such as local inflammation, injury to adjacent anatomical structures, implant fracture, loss of stability, or challenges in attaching elastic chains due to interference from surrounding soft tissues. The prevention and management of such complications will be addressed.

## Simple Summary

Orthodontic implants (OIs) are titanium screws inserted into the maxilla or mandible to serve as anchorage for moving teeth, which are often confused with dental implants. This article will provide a comprehensive comparison of the two types of screws used in dentistry, and three representative cases illustrating indications for the use of OIs in veterinary dentistry. Frequently, OIs are used to move the lower incisors lingually or to move the upper canines backwards. Moreover, their use extends to palate surgery, with screws used to hold a custom-made shield in place. This article will cover topics regarding the selection criteria, the best sites for insertion, the techniques for insertion and removal, and their possible complications, along with the prevention and management of said complications.

## 1. Introduction

Malocclusions in veterinary dentistry are frequently encountered [1,2,3,4], and owners are increasingly requesting orthodontic treatments. Head conformation impairments in dogs can be classified into three groups: mesocephalic, dolichocephalic, and brachycephalic [5]. The mesocephalic conformation forms the reference group, as this conformation is encountered in wildlife and is considered normal, even in humans. Dolichocephalic heads are long and narrow, as in Greyhounds or Border Collies, and brachycephalic breeds, the most representative being the French Bulldog, typically have short and broad muzzles, and often exhibit a high incidence of dental pathologies. In mesocephalic dogs, the maxilla is slightly larger than the mandible, with the maxillary incisor teeth overlapping the mandibular ones. Other occlusal keys are present in canines, where the tips of mandibular canine teeth are located midway between the third incisor tooth and the maxillary canine tooth. The maxillary premolars are buccally situated, presenting interdigitation, with the maxillary teeth occluding rostrally to the mandibular ones. The maxillary fourth premolar occludes on the buccal side of the mesial cusp of the mandibular first molar, and the molars have occlusal surfaces, which favors the crushing of food [5]. Human dentistry recognizes three classes of malocclusion, as classified by Edward Hartley Angle, who is considered the father of modern human orthodontics [6]. Class I refers to eugnathic occlusion, described as the reference occlusion in mesocephalic heads, and refers to a mesiodistal relationship between the first molars in humans, or carnassials in veterinary dentistry. Malocclusions occur in varying forms; thus, Dewey [7] and Anderson [8] further categorized Class I into five types of malocclusions:

1. Dewey type 1—incisors crowded and/or canines lingually positioned;
2. Dewey type 2—incisors protruding;
3. Dewey type 3—edge-to-edge contact and/or anterior crossbite;
4. Anderson type 4—uni-/bilateral posterior crossbite;
5. Anderson type 5—mesioversion of the first permanent molar (the occlusal key for human orthodontics) due to premature extraction of deciduous teeth.

Class II refers to mandibular distocclusion, where the mandible is retrognathically positioned to the maxilla [9]. Class II is also divided into two subclasses:

1. Type 1—the maxillary central incisors are palatally inclined and may be overlapped by the maxillary lateral incisors.
2. Type 2—a deep overbite and a broad maxillary arch.

Class III refers to mandibular mesiocclusion, where the mandible is situated anterior to the maxilla [10], and is divided into three classes:

1. Type 1—the dental arch is abnormally shaped;
2. Type 2—the mandibular teeth are lingually oriented;
3. Type 3—the maxillary teeth are palatally oriented palatally.

For veterinary dentistry, the AVDC has established categories for symmetrical and asymmetrical skeletal malocclusions [11]. There are three classes for symmetrical skeletal malocclusion: Class I, referring to eugnathic occlusion, but with some teeth rotated or malpositioned, Class II, referring to mandibular distocclusion; and Class III, referring to mandibular mesiocclusion. In cases of no apparent discrepancy between the jaws, the symmetrical skeletal malocclusions in these three classes are known as dental malocclusions. Asymmetrical skeletal classes are recognized under Class IV malocclusions and include maxilla–mandibular asymmetry in the rosto-caudal, side-to-side, or dorso-ventral directions.

Orthodontics in veterinary dentistry typically addresses Class I dental malocclusions, characterized by malpositioned incisor and/or canine teeth. Conventional orthodontics involves the movement of teeth with the support of adjacent teeth or with the use of extraoral devices [12]. Extraoral anchorage has historical significance in human dentistry, but it is not applicable in veterinary dentistry. Every tooth movement requires an anchorage that enables correct tooth movement and must not be displaced during orthodontic treatment. In veterinary dentistry, however, the surgeon cannot control the behavior or biting force of the animal, so reliable approaches to achieve these goals are needed.

OIs are endosseous devices designed to provide stable skeletal anchorage in orthodontic treatment. Although they do not have a unique definition in the literature, they are synonymous with mini-screws, mini-implants, and micro-implants [13,14]. The ISO 16443 (Dentistry—Vocabulary of Oral Implantology) [15] specifies terms and definitions for dental implants, instruments, and accessories, as well as the most commonly used clinical terms in the field of dental implantology. According to its definitions, a DI, or dental implant, is defined as a device primarily designed to be placed within, through, or upon the bones of the craniofacial complex to support and to resist displacement of a dental prosthesis, replacing missing teeth. An OI, by contrast, is a device specially designed to be placed through the bones of the craniofacial complex with the primary purpose of providing anchorage for an orthodontic appliance [16]. Unlike conventional orthodontic mechanics, which depend on the support of adjacent dental units and may consequently result in undesired tooth movement or anchorage loss, OIs offer a direct and predictable alternative. Thus, local anatomical conditions accompanied by insufficient dental support for stable orthodontic anchorage are indications for the use of OIs. In human orthodontics, they offer a reliable anchor in edentulous areas or in cases of periodontal disease. In veterinary dentistry, periodontal disease is less likely to be present, as orthodontic treatment typically occurs at an early age. While the utility of DIs in small animals is still questioned, OIs are beginning to be used more frequently. Kanomi [17] designed titanium OIs with a diameter of 1.2 mm and a length of 6 mm, which first entered the market in 1997. Titanium was chosen to enhance the anchorage, as it was widely used for DIs due to its biocompatibility and ability to prevent infections [18].

Palatoschisis in cats and dogs is a life-threatening condition with a multifactorial etiology [5,19]. Several surgical techniques have been described for closing this palatal defect in correlation with the following dimensions: narrow congenital hard palate clefts may be rectified with the medially positioned flap technique [5,20,21], and for wider hard palate clefts, the overlapping flap technique ensures tension-free closing of the palatal defect [5,22,23]. The hard palate may either be addressed in one session together with the soft palate, or the two procedures may be undertaken separately. When the palate defect is wide, the lack of bony support may be challenging for flap positioning, threatening its survival. Moreover, as a cat’s tongue has numerous filiform papillae [24], it may scrape the flap where there is no underlying bony support, which may compromise the surgical results. Given these aspects, the authors designed a customized shield to protect surgical flaps from abrasions caused by tongue papillae. This custom-made shield was held in place with the help of four OIs. The materials and methods will be described and discussed later in this article.

The literature on human orthodontics is abundant on this topic; however, veterinary dentistry lacks information. Thus, the aim of this article is to present retrospective case series with OIs’ indications, techniques, characteristics, and complications—and their prevention and management—regarding the use of OIs in veterinary dentistry, placing it within the context of findings from human dentistry.

## 2. Materials and Methods

Twelve cases were selected in which OIs were used for orthodontic reasons in dogs, and one case involved the use of OIs to protect a flap used in palatoschisis correction in a cat. Inclusion criteria for the cases for malocclusion were class I malocclusion. All patients had permanent dentition, with ages ranging from 5.5 months to 26 months with a median age of 10.95 months. Gender was not considered a determining factor. Mandatory criteria for inclusion in the study were a proper medical condition to undergo safely multiple general anesthetics, the owner’s willingness, as evidenced by signed informed consent, a discrepancy between the maxilla and mandible of less than 5 mm, and neutered patients. A thorough preoperative medical exam and preoperative laboratory tests, including hematology, serum biochemistry, and blood coagulation, confirmed a proper medical condition. Every selected orthodontic case had an ASA physical status of I. Premedication was performed with medetomidine (0.005 mg/kg i.m.) and methadone (0.5 mg/kg i.m.). Every patient, without exception, received an intravenous catheter through which propofol (2 mg/kg i.v.) was used to induce anesthesia. Endotracheal tubes were used to administer inhaled anesthetics. MAC for isoflurane was 1.3%. Monitoring during orthodontic procedures included evaluation of cardiac rate and rhythm, oxygenation, respiratory rate and rhythm, body temperature, anesthetic depth, capillary refill time, oral mucosa color, blood pressure, and analgesia. Local anesthesia was obtained with Lidocaine 2% (5 mg/kg) for a caudal mental nerve block for OIs applied on the mandible or an infraorbital nerve block for OIs applied on the maxilla.

Exclusion criteria were other malocclusions than rostral crossbite, rostral level bite, or mesialized maxillary canine tooth; ASA physical status other than I; non-cooperant owner; or refusal to neuter the patient.

In each case, extraoral photographs were captured from the frontal and both lateral views, along with intraoral photographs at the beginning of the treatment, during and after. The images were taken using a Huawei Mate 20 Pro, (Huawei Technologies Co., Ltd., Shenzhen, Guangdong, China) with the Macro function enabled, and a Smile Lite MDP1 (Smile line, St-Imer, Swizerland) circular light device, specifically designed for accurate dental photography. Orthodontic cases began with taking dental impressions of both dental arches using condensation silicones. The dental laboratory then scanned these impressions, leading to the creation of orthodontic devices through Computer-Aided Design (CAD) and Computer-Aided Manufacturing (CAM), Exocad 2023. To facilitate tooth movement, metallic crowns on the incisor teeth were designed with hooks on the lingual part and inclined planes on the incisal parts. These hooks were connected to the orthodontic implants using elastic chains, which needed to be changed every two weeks. At the end of the treatment, the orthodontic devices were cemented in place and removed using tungsten cylindrical burs and ultrasonic scalers.

Osstem produced the OIs used in the procedure and were manufactured in Busan, South Korea. The surgical kit utilized for implant insertion was provided by the manufacturer and included a pilot drill, hand drivers of varying lengths, handpiece drivers of different dimensions, and heads designed to fit various shapes of OIs.

All OIs were placed without angulation with the hand driver after pilot drilling. In eleven of these cases, OIs were used to lingualize the mandibular incisor teeth, and in one case, the OIs were used to distalize a maxillary canine tooth. All of the dogs included in this study were neutered. Eight cases presented a rostral cross-bite, and three cases presented a rostral level bite. Two cases, one for each indication, were selected to illustrate the use of OIs in dogs for orthodontic purposes, with the third case presenting the use of OIs for protecting the flap in palatoschisis management in a four-month-old cat, thus enlarging the range for OI indications.

OIs insertion involved the following steps:

1. Dental X-ray of the mandibular premolar teeth to determine the precise localization of the OI.
2. Regional nerve block.
3. Pilot drilling in cortical bone.
4. Dental X-ray.
5. Insertion of the OI.
6. Final dental X-ray.

Dental X-rays were obtained using phosphor plates, which allowed images to be transferred to a computer via a scanning device (indirect digital radiography). The size of the plates used was either 2 or 4, depending on the breed and dimensions of the head. Both parallel technique and bisecting angle technique were utilized during the X-ray process. Dental X-rays were taken to accurately determine the diameter of the OIs, localize them precisely, verify their correct placement, and ensure that the OIs did not interfere with the dental roots.

Every case was checked visually/taking pictures every two weeks, and if needed (more tooth movement required), elastic chains were changed under general anesthesia. A four-month-old cat was presented with a wide congenital cleft of the hard palate (palatoschisis). Firstly, surgery was performed on the hard palate, and after six months it was performed on the soft palate. In the surgical approach for the hard palate, the overlapping technique was used. A dental impression was taken with condensation silicone. A model of polymethylmethacrylate (PMMA) was made immediately and isolated with medical Vaseline. On the model, a 1:1 PMMA plaque was made on the entire hard palate. The protection shield was applied at a distance of approximately 2 mm from the flap to avoid pressure to the flap, and it was carefully polished. Four holes were made in the corners using a diamond bur with a diameter of 1.4 mm, allowing for OI placement. OIs that were 1.4 mm in diameter and 10 mm in length were inserted to protect the flap. As the OI heads were button-headed, the surgeons applied a colored photopolymerized composite on them in order to avoid hurting the tongue and to allow regular feeding. The OIs with the PMMA plaque were removed two weeks later.

OI removal is a straightforward procedure, made under general anesthesia, and does not require local anesthesia. OIs are removed by twisting them in the direction opposite to insertion with the same screw that were inserted.

## 3. Results

### 3.1. Case 1—Lingualization of Mandibular Incisor Teeth

A small-breed dog, aged five and a half months, was presented for a rostral crossbite. Preoperative laboratory tests and the medical examination revealed no contraindications for undergoing multiple general anesthetics. A classic dental impression of the dental arches was made using condensation silicones. The dental laboratory scanned the impression and proceeded with the CAD/CAM of the future orthodontic device. Both the mandibular and maxillary incisor teeth had metallic crowns with inclined planes on the incisal edges. The mandibular crowns were designed with lingual hooks to facilitate the attachment of elastic chains. The metallic crowns were cemented onto the teeth without any mechanical or chemical surface treatment—only simple drying of the crowns was performed. Two button-head orthodontic implants (OIs) were placed buccally between the roots of the mandibular fourth premolars (308 and 408). The diameter of the OIs was determined by subtracting 2 mm from both sides of the dental roots at the chosen localization between the roots. Measurements were taken from the tip of the premolar’s median cusp. For extra-small breeds, a minimal diameter of 1.2 mm is recommended, with dimensions increasing according to available bone. For a diameter of 1.4 mm, a minimum of 5.4 mm between the dental roots is required for proper insertion without risking their damage. In this case, the OIs measured 1.4 mm in diameter and 6 mm in length. They were inserted perpendicular to the mandible, without angulation. The jaw width was measured with a device to estimate the required length. After pre-drilling with the appropriate hand driver for button heads, the OIs were inserted to achieve primary stability, ensuring they were not too close to the cortical bone or exerting pressure on the soft tissues.

**Figure 1:**
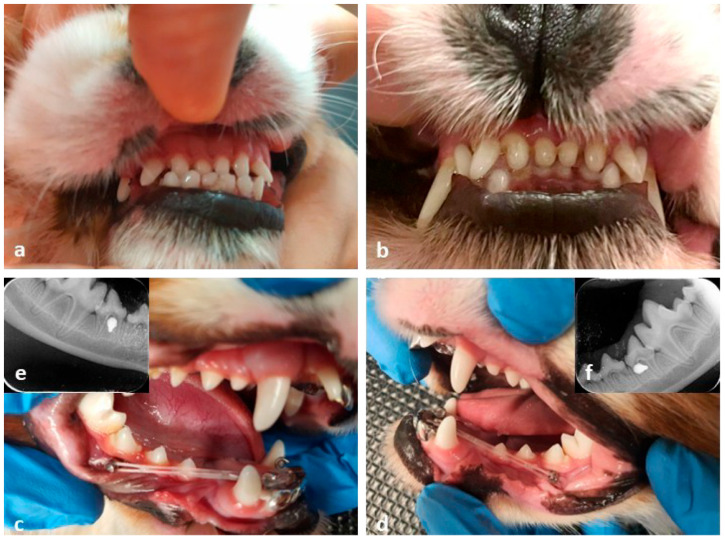
(**a**) Clinical view of the rostral crossbite. Note the inversed occlusion between 102, 101, 201, 202 and 302, 301, 401, 402, 403. The dental units were in contact. (**b**) Clinical view at the end of the orthodontic treatment. (**c**) Clinical view of the elastic chains attached to the OI—right side view. Note the lingual hooks from the lingual side. (**d**) Clinical view of the elastic chains attached to the OI—left side view. Note the lingual hooks attached to the button head OIs. (**e**) Radiological view of the inserted OI—right side view. (**f**) Radiological view of the inserted OI—left side view.

A two-week follow-up showed the occurrence of the occlusal jump and the correction of the rostral crossbite, with no need to change the elastic chains. This arrangement was maintained for two months to stabilize the results. At the end of the maintenance period, the orthodontic device was removed. A follow-up after two years revealed that the results were stable, with no changes in the color or morphology of the crown surfaces. Some aspects described are illustrated in Figure 1.

Some of the aspects described are illustrated in Figure 1.

### 3.2. Case 2—Mesially Displaced Canine Tooth in a Medium-Size-Breed Dog

**Figure 2:**
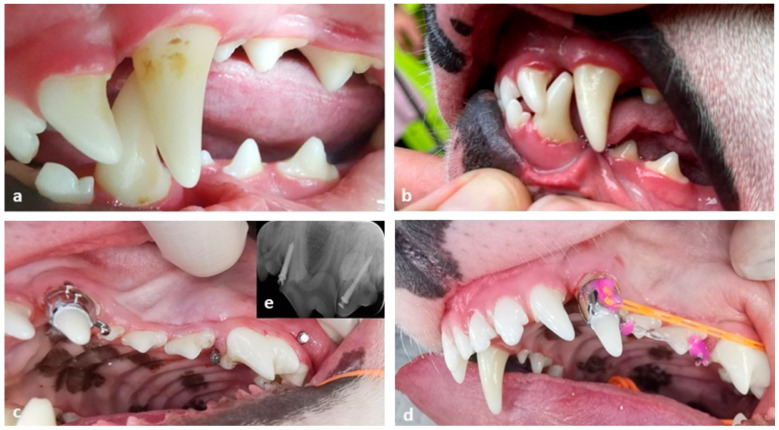
(**a**) Clinical view of the narrowed space between 203 and 204, and palatally displaced 304, impinging the palatal mucosa. (**b**) Clinical view at the end of the orthodontic treatment. (**c**) Clinical view of the orthodontic devices: a collar with two labial buttons and a distal hook, placed on the 204 and two OIs—one mesial and one buccally placed to 208. (**d**) Elastic chain placed on a labial button on 204 and colored light cured composite placed on the buttons to avoid trauma of the soft tissues. (**e**) Dental X-ray of the two OIs.

The dog exhibited lingualization of tooth 304, along with mesialization of tooth 204. In this case, considering the size of the canine root, two orthodontic implants (OIs) were placed: one in the zygomatic arch, parallel to tooth 208, and another between teeth 207 and 208 along the dental arch. The maxillary canine had a metallic collar that was one centimeter high, featuring two buttons on the labial side and one hook on the distal side of the crown. The buttons connected to the OI from the zygomatic arch, while the hook linked to the OI situated between teeth 207 and 208, using elastic chains. These elastic chains were changed every two weeks, three times. The mandibular canine required labioversion, so an orthodontic device was designed to apply forces from the lingual side to the labial side. This device in the mandibular arch consisted of two metallic collars on the mandibular canines, connected to a lingual component that included an orthodontic expanding screw. The expanding screw was activated each time the elastic chains were changed. Once there was sufficient space for the tip of the crown of tooth 304 to be positioned between teeth 203 and 204, through the distalization of tooth 204 and the labioversion of tooth 304, the occlusion of tooth 304 was normalized. All our orthodontic cases had maintenance interval of eight weeks. Several key aspects of this case are illustrated in Figure 2.

### 3.3. Case 3—Congenital Cleft Palate in a Cat

**Figure 3:**
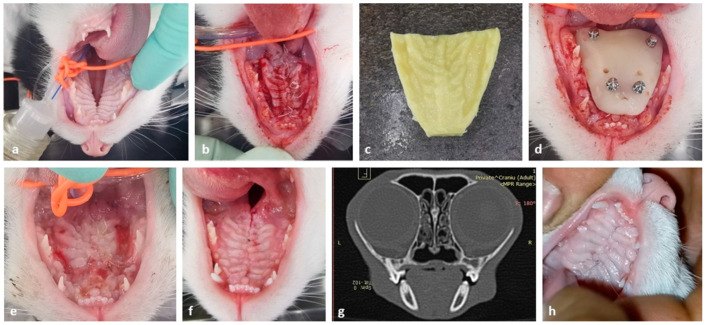
(**a**) Clinical view of the cleft palate in a four-month-old domestic short-haired cat. Note the wide palatal defect. (**b**) Intraoral view of the cleft palate treated using the overlapping technique. (**c**) Impression made with putty condensation silicone. (**d**) Protection shield applied intraorally with four OIs. (**e**) Clinical view of the surgical field at two weeks from after palate surgery. (**f**) Clinical view of the palate three months after surgery. (**g**) CT image of the hard palate two years after surgery. (**h**) Clinical view of the palate two years after surgery.

A four-month-old domestic short-haired cat was presented with large palatoschisis. Given the anatomy of a cat’s tongue, the primary concern was the post-surgery period and the abrasive effect of the tongue that would impinge on the healing process by compromising the flap. The surgical team designed a custom-made protection shield made of PMMA to be applied for two weeks, enabling primary healing of the flap. The protection shield was placed 2 mm from the flap to prevent pressure on the flap and necrosis. It was held in place for two weeks with the help of four OIs. The OIs were selected, and the 10 mm length was calculated by taking into account several factors: the width of the flap, the distance from the shield to the flap, the shield’s width, and a minimal anchor on the palatal bone. At two weeks post-surgery, the custom-made shield was removed, revealing granulation tissue, and the intact sutures. There was no presence of necrotic tissue. By three months after the surgery, the palatal mucosa was keratinized and fully healed. A two-year follow-up confirmed complete closure of the hard palate. The confirmation was obtained clinically and through a computed tomography scan. Although the scan was initially obtained to assess an inner ear pathology, it was also shared with the maxillofacial team for further evaluation. Several key aspects of the case are presented in Figure 3.

### 3.4. General Results

All OIs constituted a reliable anchorage system, facilitating precise and controlled orthodontic tooth movement. None of the cases presented infection or mobility issues, and primary stability was achieved in all cases. OIs that were used to maintain the results in the palate surgery remained in place, offering good stability for the protection shield.

None of the cases at the end of the orthodontic treatment and at two-year follow-up point showed pink or any other staining. Intra-vitam pink teeth were not associated with the use of OIs.

## 4. Discussion

The three cases presented summarize the primary indications for OIs in veterinary dentistry.

An OI has an endosseous part, a head, a transmucosal part, a body, and a tip. Classification of OIs is made regarding head design (button vs. bracket), diameter (varying from 1.2 to 2 mm), length (5 to 12 mm), body design (cylindric or tapered), transmucosal profile (from 0 to 3 mm), insertion technique (self-drilling and pre-drilling), thread orientation (clockwise or anti-clockwise), the alloy used for manufacturing them (titanium alloy or stainless steel), and sterility (sterilized or non-sterilized) [13]. The OIs used for obtaining the needed results were made of titanium, were sterilized, were button-headed, had a clockwise thread orientation, and had a transmucosal profile measuring 2 mm. The choice of design for the head was based on its scope, to ensure correct placement of the elastic chains. Moreover, the button head design was chosen as it is less traumatic to nearby mucosa. The manufacturer offered other designs, which ultimately were not chosen.

When choosing the OI, one should consider bone availability and the presence of a two-millimeter distance to nearby dental roots [25]. Dental X-rays taken before, during, and after OI insertion are taken to provide their precise location, indicating insertion sites at a distance from anatomical structures that would be damaged if OIs were inserted in them. The most commonly used sites in veterinary orthodontics are situated between the mandibular premolar roots, in the buccal side. If OIs are inserted lingually, not only is the OI insertion technique highly complex but also traumatic injury of the tongue and underlying tissues can occur. The length and diameter of an OI must correspond to the available bone volume. Thus, smaller breeds require OIs of smaller lengths and diameters than larger breeds, and each case should be approached individually.

As OIs are sometimes confused with DIs and lack precise differentiation, the authors have synthesized the differences between these two dental devices in Table 1 [15,26,27,28].

Primary stability (PS) in OIs refers to mechanical stability immediately after OI insertion. OIs are only measured via PS, by attempting to move the OI laterally with a tweezer, during which the OI should not exhibit any movement. By contrast, DIs are measured via the Implant Stability Quotient (ISQ), which can be measured at every moment of a DI’s life [4,29,30]. PS depends on various factors, including bone density, cortical width, thread design, and diameter and length of the OI, as well as a correct and clean insertion technique [16]. If the OI presents mobility, it should be removed, as it does not offer reliable stability for tooth movement. Inserting the OI perpendicularly into the cortical bone usually ensures the PS is needed.

Increasing the torsional force during OI insertion may lead to bending or fracture of the OI [16]. Kim et al. [31] investigated the issue of whether to insert OIs via drilling or not, as well as the relationships between OI dimensions and PS, and long-term results and complications. Even though PS is increased if an OI is inserted without drilling, studies recommend the use of a pilot drill [32]. The risk of OI fracture is very high if inserted without pre-drilling, with OIs usually fracturing at the least resistant part, at the joint between the head and body in the cervical region [32]. If the site is not predrilled, the OI can be displaced into the soft tissues, which complicates its removal. When predrilling, it is necessary to drill in a steady, correct direction, which is, in a great majority of cases, perpendicular to the cortical bone. Otherwise, if the hand moves while drilling, the resulting cavity will be larger, compromising PS when inserting the OI. Pilot drilling is also reported to reduce microdamage to the cortical bone [15,32].

Additionally, excessive OI loading can induce osteolysis and lead to OI mobility.

Another essential aspect is the placement level of the OI head. It is necessary that the head remains above the soft tissue and does not penetrate the bone or come into close contact with it. Otherwise, if these complications occur, it then becomes difficult to insert elastic chains and keep them in place. Regarding the distance from the nearby teeth, 2 mm of bone should be present between the OI and the teeth [31].

Removing OIs is not a complicated procedure; however, the surgeon must know the orientation of the threads, as there are different systems containing threads oriented to the right or to the left. Thus, the thread orientation should be noted in the medical records so that when removing the OIs, high force should not be applied, thus avoiding fractured OIs.

Management of a fractured OI typically involves incision and small-bone drilling around the OI to facilitate extraction. The authors do not recommend leaving a fractured OI in place, as it may cause a foreign body reaction.

Another complication that may arise with regard to OIs is mucosal inflammation. In our study, inflammation in cases of OIs in small animals was minimal and resolved after the OIs were removed.

Regarding the use of OIs in patients with periodontal pathology, one should pay attention to the force applied, as the alveolar bone is smaller in volume in patients affected by periodontal disease. Applying pressure to the affected periodontal ligaments and alveolar bone may lead to gingival recession, dental mobility, or even tooth loss [31]. It is important to note that in human dentistry, periodontal therapy precedes orthodontic treatment. Only after achieving the necessary health status of the periodontal structures and maintaining the results for a specified period is the orthodontic treatment initiated. If oral hygiene is poor and the results of periodontal therapy are weak, orthodontic treatment should not be initiated. However, in veterinary dentistry, this rule is hard to implement. The animal lifespan is shorter, and all procedures are performed under general anesthesia. If periodontal treatment with guided bone regeneration is performed in small animals before orthodontic treatment, the results remain questionable, and any periodontal problems are enhanced by abnormal forces applied to the periodontal ligaments due to malpositioned teeth. Plaque accumulation is favored by malpositioned teeth [33,34], making self-cleaning harder to manage. Nevertheless, the principle in human orthodontics to achieve periodontal stability before initiating orthodontic treatment [35] does not apply in veterinary orthodontics; thus, a small animal patient with periodontal issues is excluded from orthodontic treatment.

Other complications associated with OI insertion have been widely documented in human medicine [36]. One significant complication that can be prevented is damage to the dental roots. By carefully examining and calculating the dimensions via dental X-rays, one can insert an OI precisely without damaging the roots, thereby avoiding potential negative consequences in the long term. In human dentistry, pink staining due to internal resorption that occurs in conjunction with orthodontic treatment has been reported [37]. In veterinary dentistry, the appearance of an intra-vitam pink tooth was reported as a result of a DI insertion in a dog [38]. Human studies have documented that pink staining implies higher insertion forces when the OI is inserted into the roots compared to OI insertions not involving any root [39,40]. Radiographs taken before and during the procedure associated with the 2 mm rule can serve to ensure an insertion without damaging the nearby dental units. In our study, none of the cases treated with OIs at the end of the orthodontic treatment showed any staining of the dental crown.

Regarding the perforation of the nasal floor in the presented case of the cat, the healing was spontaneous without any oronasal fistula. The length of the OIs was 10 mm; the plate had a width of 2–3 mm and was placed at a distance of 2–3 mm from the flap. The flap was approximately 3 mm thick, and the nasal cavity was not perforated. Each OI was only inserted in the cortical bone of the palate, with the distribution of the four OIs ensuring stability of the protection shield. Similarly, human dentistry has reported spontaneous healing of the maxillary sinus in cases of perforation smaller than 2 mm [41].

A traumatic soft tissue injury to the buccal adjacent mucosa, which appeared as a kissing ulceration, was self-limiting and resolved by applying a colored-light-curing composite on the head of the OI without bonding. Thus, the composite was easily removed, and the head of the OI was not damaged during the change in elastic chains. This protocol, which utilizes light-curing materials, was successfully implemented in human dentistry [42].

Custom-made palatal shields have been used in human oro-maxillo-facial surgery to protect the newly restored palate and are maintained in place without any OI, by being applied and held in place by the adjacent teeth [43]. However, the risk of losing the shield by anchoring it to the cat’s teeth was high, given that the cat was in the stage of transitioning from temporary to permanent teeth. Therefore, the authors considered maintaining the shield with the help of OIs. The OIs provided adequate protection, and healing occurred without any fistulae. A two-year follow-up of the case revealed the growth of the palatal bone, as seen in CT findings. Regarding the palatal growth that follows surgery, the authors report findings similar to those of Wright et al. [44], who reported spontaneous regeneration of a mandibular body after a mandibulectomy. The ages at which the surgeries were performed were similar, and the finding that the palatal bone continued to grow following cleft palate surgery confirms that even in the maxilla, the developing head induces bony formation where it initially lacked it. The maxilla continued its development, and no malocclusion developed as a result of cleft palate surgery repair.

The authors did not encounter any alveolar bone exostoses as reported in human dentistry [45,46].

Distalization of the maxillary canine tooth in one case in our study required the use of two OIs. Canine teeth are positioned in a transitional site on the dental arch, being located on the curve that reunites the lateral parts of the dental arch with the line of incisor teeth. The forces exerted on canine teeth are high, as noted by Zhou et al. [47]. Thus, their root morphology has adapted to high forces, resulting in noticeable volume. High orthodontic forces need to be applied to the canine tooth to be distalized as a result. This is the reason why the authors preferred to use two OIs to manage canine tooth distalization.

Regarding ethics in veterinary orthodontics, all dogs were neutered and removed from breeding if they were purebred [5,48]. The orthodontic treatment was carried out for functional reasons rather than cosmetic ones. Misaligned teeth can retain food, leading to increased dental plaque accumulation and a higher risk of periodontal diseases occurring earlier than in patients without dental issues. Moreover, conditions such as level and cross-bite are traumatic occlusions that can result in pathological attrition and complicated crown fractures over time, causing significant pain and discomfort [5,48]. The authors adhere to the ethical guidelines of the American Veterinary Medical Association (AVMA). The first principle of these guidelines emphasizes animal welfare as the top priority when using orthodontic treatments in small animals. The medical team was honest and transparent with the pet owners, presenting all potential complications that could arise during orthodontic treatment and ensuring informed consent. They clearly explained the potential benefits, justifying the associated risks and costs equitably [49]. The veterinary team implemented the best available procedures, which were imported from human medicine. It is widely recognized that any new medical procedures authorized for humans should first be thoroughly tested on animals, in alignment with the one health concept. Additionally, with a human dentist who is also a final-year veterinary medicine student serving as a consultant, the authors believe that the patients benefited from the most recent and validated medical techniques. The pain developed during orthodontic treatment with OIs was not noticeable and did not require any medication to be discontinued. The risk of nasal perforation in the third case was present, though a precise measurement ensured the cortical maintenance for OIs. Regarding the potential interference with craniofacial growth during the two-week maintenance of the custom-made palatal shield, the authors considered the risk to be minimal. Their hypothesis was later supported by the normal development of the head and the Class I relationship observed at the carnassials level on CT images taken two years after the surgery.

## 5. Conclusions

All cases involving the use of OIs had a successful outcome, without impacting dental and periodontal tissues. The good results can be attributed to strategic case selection and a more manageable number of cases, ensuring a higher quality of outcomes. The use of OIs may be a reliable solution for tooth movement in veterinary dentistry.

The innovative, custom-made PMMA shield, maintained with four dental OIs, may offer a reliable solution for the post-operative healing period when a surgical flap is exposed to the mechanical pressure applied by the tongue and its papillae.

## Figures and Tables

**Table 1:** The main differences between OIs and DIs.

Characteristics	OI	DI
1. Purpose	Anchorage	Replacing missing tooth
2. Time in the oral cavity	Limited period of time/months	Extended period of time/years
3. Osteointegration	Not necessary	Mandatory
4. Dimensions	Smaller	Larger
5. Diameter	1.2–2.0 mm	3 to 7 mm or more
6. Length	5–12 mm	6–25 mm
7. Coronal part	Button/Bracket	Cylindrical/conical form
8. Localization	Outside the dental arch	On the dental arch
9. Associated inflammation	Minimal local inflammation	Periimplantitis
10. Stability measurement	Approximation	Precise measurement/ISQ
11. Types of stability needed	Primary stability needed	Every moment of a DI’s life needed
12. Acceptance in veterinary dentistry	Accepted	Questionable
13. Surface treatment	None	Yes
14. Price	Low	High

## Data Availability

The original contributions presented in this study are included in the article. Further inquiries can be directed to the corresponding author.

## Abbreviations

The following abbreviations are used in this manuscript:

OI	orthodontic implant
OIs	orthodontic implants
DI	dental implant
PS	primary stability
ISQ	implant stability quotation
